# Experiences of homeless women on maternity health service utilization and associated challenge in Aksum town, Northern Ethiopia

**DOI:** 10.1186/s12913-019-4201-3

**Published:** 2019-06-06

**Authors:** Hailay Gebreyesus, Abebe Mamo, Mebrahtu Teweldemedhin, Berihu Gidey, Znabu Hdush, Zewdie Birhanu

**Affiliations:** 1 Department of Public Health, College of Health Sciences, Aksum University, Aksum, Ethiopia; 2 Department of Health, Behavior and Society, Institutes of Health, Jimma University, Jimma, Ethiopia; 3 Department of Medical Laboratory Science, College of Health Sciences, Aksum University, Aksum, Ethiopia; 4 Department of Public Health, Mekelle University, Mekelle, Ethiopia

**Keywords:** Experience, Homeless women, Maternity health service, Utilization

## Abstract

**Background:**

Homeless women are a highly vulnerable group for risks of pregnancy and childbirth-related complications. They may also face multiple challenges to access and utilize maternity healthcare services. This study was aimed to explore the experience of homeless women on maternity healthcare service utilization and associated challenges in Aksum Town, Northern Ethiopia.

**Methods:**

An exploratory qualitative study was employed using in-depth interviews among 22 study participants from February to March 2016. Purposive sampling was used to recruit 12 homeless mothers who gave birth when being homeless in the last 12 months and 10 healthcare providers as key informants. Data were captured using audio recorders and field notes and transcribed, translated verbatim and thematic analysis approach was facilitated using ATLAS.ti7 software.

**Results:**

The finding reveals that homeless women did not use any of the basic maternity health care services, namely antenatal care, skilled birth attendance, and postnatal care. Lack of permanent place and awareness, and fear of stigma and discrimination were some reasons hindering homeless women from using the services.

**Conclusions:**

Even though maternity health service utilization is the most crucial intervention to reduce maternal and newborn deaths, this finding shows that maternity health service utilization among homeless women was limited. Socio-cultural, socioeconomic and healthcare-related factors contributed to the non-use of these services. Efforts should be made to address the challenges faced by homeless women to utilize maternity health services.

## Background

Homeless refers to a social category of people who are living without a regular and permanent residential house [1]. Homelessness may be due to unemployment, poverty, war, political unrest, natural disasters, forced removal and also personal factors such as mental disorders, substance abuse, domestic violence, and family disagreement [2]. Majorities of them live in urban areas, and they represent a rapidly growing population at risk for poor health outcomes [1, 2].

Pregnancy and childbirth-related complications remain the first cause of maternal deaths and disability worldwide; there were an estimated 289,000 maternal deaths in 2013 [3]. Beyond the lack of a safe and suitable home, homeless women’s are exposed to substance use, poor nutrition, unintended pregnancy, stress, and lack other necessities [4, 5]. They face higher risks of pregnancy and childbirth-related complications as compared to the general population of women [6–8]. They are also a highly vulnerable group for maternal morbidity and mortality as compared to the general population of women [9].

Even though homeless women are a highly vulnerable group for risks of pregnancy and childbirth-related complications in developing countries including Ethiopia, they may also face multiple challenges such as financial difficulties, lack of social support and negligence to access and utilize the basic maternal health care services [10, 11]. Antenatal, skilled birth attendance and early postnatal care services are amongst the major interventions aimed at reducing maternal and newborn deaths worldwide but they are highly stratified by poverty and other social determinants of health [12–14]. This indicates that homeless women have limited access to maternity health services due to lack of permanent residence, [11, 15–17], lack of access to information, and lack of social support [5].

In Ethiopia, pregnancy and childbirth-related complications are among the top causes of maternal mortality with an estimated 676 maternal deaths per 100,000 live births; which is the highest figure in the world [18]. Skilled attendance during pregnancy, delivery and postnatal period is the most appropriate cost-effective and achievable strategies particularly in resource-poor countries to reduce maternal and newborn deaths [19]. However, skilled attendance in Ethiopia is still very low; about 40, 15 and 12% of the women utilize antenatal care, skilled birth attendance, and postnatal care services respectively, while 5% of births are unattended [20].

The key barriers for low maternity health services utilization are poor approaches of health care professionals, women’s educational status, lack of exposure to media, inadequate household income, problems during a pregnancy, childbirth and postnatal care, unwanted pregnancy, and previous maternity experiences [21–26]. Therefore, homeless women are experiencing unaided delivery outside a health care facility-due to their difficult living conditions [11, 17]. This is often due to social marginalization and discrimination by both health care providers, and the health care system [27–33].

Several studies have been done on maternal health service among the general population of women at the local and national level [9, 12, 25, 27, 34] but none of this addressed the lived experience of homeless women on maternal health care service utilization who are economically, socially and nutritionally disadvantaged. Therefore, this study was aimed to explore the experience of homeless women on maternity health service utilization and its challenges using an exploratory qualitative study design in Aksum Town, Northern Ethiopian.

## Methods

### Study setting and period

Aksum town is one of the ancient towns in Ethiopia, found in Central Zone of Tigray Regional state and located 1024 km North of Addis Ababa with an altitude of 2189.91 m above sea level. The town has an annual average temperature of 18.3 °C, annual rainfall of 652 mm and an area of 3247squere kilometer. According to the town’s administrative office report of 2016, Aksum town has about 63, 435 populations, of which 32,698 are female. Because Aksum is a historical place many local and foreign tourists visit the town every time. To use this advantage, there are many homeless peoples in the town coming from different parts of the country; engaged in begging around the historical places including the churches. In the town, there are two public health centers, one general hospital, one referral hospital, and four private clinics potentially fit to provide maternity healthcare services. Considering the season for the high number of homeless women in the surrounding the study was conducted from February 1, 2016, to March 4, 2016.

### Study design

An exploratory qualitative study was conducted inductively.

### Population

All homeless women living in Aksum town in the last 12 months and who gave birth when being homeless in the last 12 months; thus, homeless women were purposely selected and interviewed to understand their experience as per the study objectives. In addition, key informants (health care providers) were purposely selected to gather the information that complements women’s data. Women’s and key informants who were unable to communicate due to physical or mental illness were excluded from the study.

### Sample size and sampling techniques

Twelve homeless women and ten health care providers (key informants) were interviewed; further sampling process was stopped based on the saturation of coming ideas. Six of the key informants (2 medical doctors, 2 midwives, and 2 nurses) were from the governmental public hospitals and four of them (2 health officer, and 2 midwives) were from the public health centers. We used local guidance to locate and identify potential sampling sites where homeless women actually live and concentrate such as churches, main roads and around other public institutions. Criteria based purposively sampling was used to select participants. Participant recruitment was through a direct approach. Mothers who fulfill the inclusion criteria participated in the study. Key informants (healthcare providers) were also enrolled in the study based on their experiences and involvement in the provision of maternity healthcare services providing and their willingness to participate in the study with the help of a respective head nurse.

### Data collection method

Semi-structured interview guides for homeless women and healthcare providers were developed by investigators. The guide was first developed in English, translated into Tigrigna (the local language) then back-translated and rechecked by a third person, to ensure its consistency and correctness. The interview guide contained open-ended questions with four key items; socio-demographic characteristics of the participants, experience of maternity health service, reasons for not seeking maternity health service (ANC, SBA, and early PNC), and what constraints they faced during pregnancy, childbirth and postnatal care.

Key informants were asked about where homeless women accessed maternity health service, what challenges encountered during pregnancy, childbirth and postnatal care services and suggestions for improving the provision of maternity health service to homeless women. Probing questions were also asked, as needed to get a more in-depth understanding of the participants’ feelings and their experience with the situation.

Once the respondent was identified, a suitable and a private place was arranged based on their interest to conduct the interview with each respondent. All interviews were prescheduled and took place in rooms and healthcare facility offices that guarantee optimum privacy. The interviews were conducted in the local language, Tigrigna supporting interview guide, recorders and also wrote field notes. The interviews ranged from 45 to 60 min per participant. All participants agreed to be audio-taped. After each interview, notes including memos of participant behavior and contextual aspects were taken to assure triangulation of the data with the record.

### Data analysis

We analyzed the data simultaneously with data collection. All interviews which were audio-taped and field notes of the interview were fully transcribed verbatim to Tigrigna (the local language) then translated into English after careful reading, listening to the audio and field notes independently. The individual transcribed documents were imported into ATLAS.ti7 computer software program as a separate primary document in a new hermeneutical unit for coding and analysis. After reading and re-reading the transcribed document line by line, the raw data were systematically coded and categorized to themes and sub-themes. Then, we created non-repetitive central themes that were constructed based on the natural meaning of categories. Finally, we cross-cheeked the themes that emerged after analysis; likewise, in the overall process of this data analysis, an inductive approach was implemented to identify themes and sub-themes.

### Data quality management and insurance

We considered a different set of criteria focusing on the credibility, dependability, transferability, and conformability of the study using different techniques. Per-test was conducted in a similar setting and participants but out of the study area, edited and modified to our setting by an expert in the maternity health field. We invited some healthcare providers who were participated in the actual interview to review the findings and ideas which they think if they correctly represent their point of views were taken for the study. The collected data from homeless women and healthcare providers were triangulated during analysis to increase the credibility of the findings. Respondent bias and the risk of reactivity whereby holding back researchers preconceived ideas about the issue under study. Moreover, public health experts from different experiences were used to check the consistency between the analyzed data and the final textual findings.

## Results

The age of homeless women who participated in the study ranged from 25 to 44 years, with the mean (±SD) age of 33.6 ± 4.52 (Tables 1 and 2). The participants of the study were a composition of 5 divorced, 4 married, and 3 widowed women. Most participants (8 out of 12) were unable to read and write. Out of the 12 participants, 9 participants gave their recent delivery outside a health facility whereas 3 participants delivered at the healthcare facilities.

**Table 1:** Background information of research participants from homeless women, February to March 2016 (*n* = 12)

Characteristics of participants		Frequency
Age	25–29	2
	30–34	5
	35–39	3
	40–44	2
Marital status	married	4
	divorced	5
	Widowed	3
Duration of homelessness	1–5 years	7
	6–10 years	4
	Above 11 years	1
Educational status	Illiterate	8
	Primary education (1-8grade)	3
	Secondary (9-12grade)	1
Number of children	2	8
	3	2
	4	2
Place of recent delivery	outside a healthcare facility	9
	at healthcare facility	3
Employment Status	Unemployed (begging)	12

**Table 2:** Background information of key informants involving in in-depth interview from different public health facilities in Aksum, February to March 2016 (*n* = 10)

ID	Sex	Age	Profession	Level of Education	Position	Experience (Year)
HCP1	Female	30	Health officer	BSc	Practitioner	5
HCP2	Male	35	Nurse	BSc	Nursing head	3
HCP3	Female	40	Midwifery	BSc	Provider	6
HCP4	Male	32	Midwifery	BSc	Provider	8
HCP5	Male	45	Medical doctor	MD	Practitioner	4
HCP6	Female	36	Midwifery	BSc	Provider	7
HCP7	Male	37	Medical doctor	MD	Practitioner	8
HCP8	Male	32	Health officer	BSc	Practitioner	4
HCP9	Female	31	Nurse	BSc	Head nurse	6
HCP10	Female	38	Midwifery	BSc	Provider	5

Note: *HCP* health care professional

The participants stated that their time of homelessness ranged from 1 to 11 years with a mean of 5.25 years and the reported number of children ranged from 2 to 4 children. The background characteristics of key informants are also presented in Table 2 in detailed.

### Maternity health care service experience

In this study, homeless mothers did not get the most important maternity care services during their pregnancy, delivery and after delivery. Homeless mothers who did not utilize antenatal care, postnatal care services, and who delivered their children outside the healthcare facilities suggested different reasons. The three core themes mentioned by the participants as barriers are previous experience and perceptions, social and economic issues, and cultural beliefs. The subthemes under previous experience and perceptions were lack of awareness and permanent place and previous maternity health care services. The subthemes under social and economic issues were fear of stigma and discrimination, the poor approach of health care professionals and lack of social support. Finally religious and traditional believes were subthemes under cultural beliefs. The core themes and their subthemes are presented in detail with appropriate descriptions below.

### Lack of awareness

The findings indicated that there is a lack of information among homeless women about maternity care services. Homeless women did not know where and when they should seek maternity care services, and they were not aware of the importance of maternity care services during their pregnancy, delivery and after delivery. Moreover, they did not know the potential risks of pregnancy and childbirth. They overlooked the role of maternity care services simply dictates that “maternity care service is only recommended for richest women as luxurious”. Some of the participants stated that pregnancy and childbirth is normal and natural and they have perceived that maternity care service is costly and disadvantageous. The lack of information among homeless women was presented as follows: *“…During my last pregnancy, I did not use antenatal care services since I did not have any information about the service including the time, place, for which the service is…” (HW01: a homeless woman)* Another homeless participant strengthened this idea as follows:

“…I gave my last birth in church compound, and all my children were born out of health care facility (assisted by my relatives). Though I faced many difficulties, I did not give value to institutional delivery because I always live around the churches. Besides, I thought that health care delivery is left for wealthier mothers; I didn’t have awareness about free maternity service.”(HW06: a homeless woman)

Besides, the key informants who participated in the study noted that homeless women do not get information about the importance of maternity care services because they are not involved in maternity health related sessions due to their living conditions. Moreover, they are not exposed to mass-media like television, radio and others. All these reasons negatively affected homeless mothers’ awareness on the importance of maternity care services. The quoted idea from one healthcare provider is presented here in after:

“…From my experience most homeless mothers are not utilizing maternity care services during their pregnancy and child birth because they do not have information about the importance of the service from health care workers and other Medias.” (HW08: a homeless woman)

### Fear of stigma and discrimination

The result obtained from the interview showed that homeless women tend to hide their pregnancy from their relatives, community and health care providers. They are discriminated by health care providers and communities. The reasons for their denial were distorted self-image, shyness, and afraid of ridicule from the early stage of pregnancy. The ideas quoted from the homeless participants are presented as follows:

*“…I did not go to health care facility for antenatal care service follow up during my recent pregnancy because my relatives told me that if the health care providers know a pregnant mother is homeless, they give medications to induce pregnancy, and they reported to other bodies (police). ” (HW02: a homeless woman)* Another participant added a reason why she does not use antenatal care service as follows:

*“When I was pregnant my recent child I did not want to go health care facility because I fear health care providers’ verbal abuse or unnecessary words (‘beggar’) because I am poor*.” *(HW04: a divorced homeless woman)*

The key informants who participated in this study noted that most homeless women have a poor perception about their pregnancy; they were unwilling to share pregnancy-related issues especially for health care providers because they associate everything with their poverty. Moreover, they feel as if they do not have the right to be pregnant. The quoted response of the participants of the study is presented hereinafter:

“…*Homeless mothers have a poor perception about their pregnancy. If women disclosed their pregnancy, they perceived that they will be stigmatized by the community and health care providers. So, they are afraid of showing themselves as pregnant and seeking ANC follow-up.” (HCW01: a health officer participant)*

### Lack of permanent place

Almost all homeless women participated in the study mentioned that the lack of permanent residence was one of the barriers for not utilizing maternity healthcare services. They were inaccessible to local health extension workers, women development army and other health care providers due to their lack of permanent address and lack of identification documents in nearby health care facilities. They were mostly living in isolated places like in the compound of the church, at streets and other open spaces. Moreover, most of them were mobile from one place to another place for their livelihood activities. The directly quoted responses from the homeless mothers are summarized as follows:

“…My recent child was delivered outside a health care facility and I didn’t go to health care facilities for post-natal care services since I was outside the town for daily food.” (HW05: a married homeless woman)

Key informants who participated in this study also noted that homeless mothers do not receive community based health interventions due to their lack of a permanent home, and lack of identification document in one health care facility. The directly quoted response from healthcare providers is summarized as follows:

“...From my experience, most homeless mothers are inaccessible to deliver some of the most important maternity care services (home-to-home postnatal care service, immunization campaign, health education, etc) because they have no permanent place; they move from place to place to find their own daily foods. Consequently, they have not been receiving even the outreach maternity care services.”(HCW04: a nursing participant)

### Religious and traditional beliefs

Participants of the study shared common traditional and religious believes that hinder them from the use of maternity care services. They mentioned that St. Marry had helped them to have a safe birth. All their relatives and elder mothers who are living in the compound of the church believe in praying rather then going to a health care facility and they believed that the prey will help the mother to have an easy delivery without the need of the skilled birth attendant. Almost all participants mentioned that God protected their pregnancy and childbirth.

“…I faced painful labor for 3 days with the absence of home. However, my problem was solved with the help of God and his mother ‘St. Marry’. When my relatives prayed to God and, gave me holy water and sop mixed with White onion, my pain got lighten.” (HW09: a divorced homeless woman)

The pregnant mothers due to they are traditional and religious beliefs, use holy water, abdomen messaging using local butter, pray to God, and use of traditional medication prepared by relatives, elder mothers, and religious leaders to prevent them from evil. Some elder mothers still believe that there is an evil spirit inside the mother that will never be exorcized. Consequently, the pregnant mother always obliged to be loyal to that spirit, and she should not go anywhere for delivery. Since it is believed that the evil spirit will punish the mother for her wrong actions. The quoted idea from the participant of the study is presented as follows:

“…Pregnancy is the gift of St. Marry; therefore, I had never gone to health facilities, instead, I always use holy water and local butter for messaging my abdomen to facilitate my delivery and to prevent me from illness.” (HW02: a single homeless woman)

One health care provider who participated in this study shared the opinions of homeless mothers as follows:

“…Most homeless women in Aksum town were living around the church. Some elder mothers were also living with them, and they did not encourage visiting the health care facilities seeking maternity health service because of religious beliefs. In this regard, homeless women cannot ignore their elder mothers’ decision, and cannot express their opinion on the matter because they consider it as sinful.” (HCW03: a nursing participant)

### Previous maternity care service experience

The findings indicated that even though three participants received maternity care service during their previous pregnancy, delivery, and post-delivery. Almost all homeless mothers who participated in this study had negative experiences in their maternity care service. Homeless women who received at least one maternity care service in their previous pregnancy, delivery, and post-delivery mentioned that lack of attendance, excessive waiting times, unclear communication with health professionals, uncomfortable physical examinations, and discrimination were reasons hindering them from the use of maternity care services. Quoted idea from the homeless participants is presented as follows

*“…During my first pregnancy, I experienced severe abdominal pain, cramps and my relatives approached me to go to the hospital. Health care provider too advised me to take medications only, and they informed me to come back when my labor starts. Lately, I realized that I was actually in true labor and forced to deliver and my child died. That experience eroded my confidence and trust on health professionals as a result of which I decided to deliver at the open house for my recent child.”(HW010: a divorced homeless woman);* another homeless participant strengthened this idea as follows:

“…I was receiving postnatal care service from the health care professional immediately after the delivery of my recent child at the hospital. The nurse gave me medications; she immunized my child and advised me about postnatal care. But, I did not back to the hospital for my follow up because the vaccine was uncomfortable for my child. ” (HW012: a widowed homeless woman)

### Poor socio-economic status

The finding of this study disclosed that almost all the participants always engaged in begging and other activities to find daily food for their survival. All homeless mothers faced financial limitations to visit health care facilities for maternity care service and a shortage of other fees for services unavailable at governmental health facilities. The quoted idea from the participants of the study is presented as follows:

*“…I faced economic problems during the delivery of my recent child. People took me to a health center by their own contribution, and I was again referred to the hospital by ambulance. I delivered safely, but during discharge, no means of transport was secured. It was difficult for me to go back, and the nurses begged some birr from the hospital workers, and they gave it me.” (HW06: a homeless woman);* another homeless woman strengthened this idea as follows:

“…I spend my time caring for my children and in begging practices; my life is hand- to- mouth. Therefore, though I know the presence of a healthcare facility, I do not have time to go there. I have three children, so how could I go to the healthcare facilities leaving them alone without food?”(HW04: a homeless woman)

The key informants who participated in the study mentioned that different costs have effects on homeless mothers to use maternity care service utilization. The directly quoted idea from the healthcare provider is presented as follows:

“…Most homeless mothers come to our hospital after they have developed a serious complication which makes it difficult to manage. I think this occurred due to lack of social support because most homeless women are poor and live alone.” (HCW03: a nursing participant)

### Lack of social support during pregnancy and childbirth

The finding of this study revealed that homeless mothers experience a lack of physical, emotional and material support during their pregnancy, delivery, and post-delivery from health care providers, health extension workers, relatives and from communities at large in facilitating transportation and directing them to go to healthcare facilities to use maternity care services. The quoted idea from the participants is presented as follows:

*“…My labor started during nighttime suddenly; no one was with me during this difficult time. Though I was willing to visit the health care facility, I was unable to reach without support…no one shared my pain. The next morning, I delivered with difficult conditions, and my child died after a few hours…she was crying” (HW02 single homeless woman*). Another homeless woman strengthened this idea as follows:

*“…I cannot go to the health care facilities for ANC service leaving my children alone. After all, who can prepare food for me and my children if I go to visit health service facilities?” (HW07: a divorced homeless woman*)

The key informants who participated in the study also strengthened homeless mothers’ experience. The directly quoted idea from the healthcare provider is presented as follows:

*“…From my observation, most homeless women did not use maternity care service. This could be due to lack of assistance during their pregnancy and labor. No one is there to assist them in caring their children and while visiting a health care facility because they lack permanent neighbor, relative or cloth families near them”* (*HCW08: a participant from the health center*). Another healthcare provider from the hospital also supported the above idea as follows:

*“…Some economically poor mothers including homeless mothers come to our hospital for delivery supported by volunteers, and* they *want to stay more in the hospital. However, due to lack of beds, we do not have any other option than to discharge them immediately as other mothers.” (HCW03: a* medical doctor *participant*)

### Poor approaches of health care providers and negligence

Homeless women who experienced or heard from their relatives about poor approaches of health care providers (disrespect, unclear communication, verbal abuse, discrimination, and negligence) prefer to not utilize maternity care service. The idea quoted from the homeless participants is presented as follows:

*“…I had labored the entire day; I was terribly ill. My husband and other people took me to hospital. At the hospital, we got a doctor but he was not listening to me, and he was aggressive. The delivery was difficult; I lost too much blood. Since my child was quiet, I was trying to see her but he did not allow me to do so. Finally, he said ‘your daughter has died. I lost my child this way (…she cried)” (HW07: a divorced homeless woman).* Another homeless woman added as follows:

*“…My recent child was born outside of the health care facility; during my first labor, I experienced severe pain for 2 days. After that, volunteer people took me to a health center. However, the health care providers were not respecting me when they were taking my history. Their approach was cruel to me as compared to other mothers, and there was no special support like food, drink, and cloth. That experience eroded my trust to health professionals; as a result, I decided not to deliver in a health center, my last child.”* (*HW04: a homeless woman*)

Key informants (healthcare providers) who participated in the interview mentioned that lack of special support like counseling, advice and health education by health care providers at health care facility during antenatal care, delivery and postnatal care services and at the community were another reason for homeless mothers do not utilize. The directly quoted idea from the healthcare provider is presented as follows:

*“Some homeless mothers come to our hospital might be for maternity health services. However, they are neglected to receive services; the health care community fail to realize that homeless women are seeking services unless they are physically sick looking. Most of the time presence of homeless people in the compound of the hospital is considered as random access as being crazy or for the sake of begging.” (HCW0: a* midwifery *participant)*

## Discussion

The government of Ethiopia has introduced and is currently implementing a policy that provides free maternal healthcare services to all pregnant women and new babies in all government healthcare facilities. However, the findings of this study reveal that homeless women did not get the most important maternity care services during their pregnancy, labor, delivery, and after delivery. Lack of awareness, fear of stigma and discrimination, lack of permanent place and previous maternity care service experience, negligence and lack of assistance, religious and traditional beliefs, poor socioeconomic status and poor approach of health care professionals were found to be the core barriers to access and use maternity care services. Even though there are limited studies about maternity care services utilization and barriers among homeless women at the national and local level, the findings of this study are consistent with the previous qualitative studies carried out elsewhere [5, 14, 15, 19, 22, 24, 25, 35].

Homeless mothers were negatively affected and did not utilize maternity healthcare services possibly due to their difficult living conditions; they continuously did not have permanent residence and they might not be benefited from the home to home maternity health-related health education programs. Besides, they have no access to mass-media like television, radio, and other social media. Previous qualitative studies conducted in the United States, Malawi, Indonesia and Ethiopia also found that lack of information about the importance of antenatal care, skilled birth attendant and postnatal care services during their pregnancy, childbirth and after delivery was a key barrier for homeless women to receive very limited maternity care services at health care facilities [5, 12, 14, 22, 24]. Another study conducted in Pakistan has also shown that a lack of information to visit a health facility and lack of previous experience was among the barriers for pregnant women not to visit healthcare facilities for antenatal care and delivery services [21].

The findings of the current study also indicated that previous maternity service experiences, the poor approach of health care providers, stigma and discrimination, lack of social support and negligence were found to be the barriers of maternity care services utilization among homeless mothers. The negligence of the health care community and the social network is a driving force for social exclusion which makes homeless women has weak social support and poor service linkage [25]. A study conducted in China demonstrated that inequities in term of income level and place of residence were key barriers for pregnant homeless women not to deliver at a health facility [22]. On the other hand, poor attention or poor approaches of the health care professionals for homeless women seeking maternity services negatively affected their attitude to the health care providers [34] as well as their plan for future maternity services [24]. Skilled attendance during labor and delivery is accepted to be the most appropriate cost-effective and achievable strategy in resource-poor countries to reduce maternity and new child mortality [19]. However, the finding of this study revealed that most homeless women did not use health facilities during their delivery assuming that they will be neglected. A study conducted in the United States also supported this finding in that lack of provider-client relationship, lack of social support, fear of stigma and discrimination and poor socioeconomic status were reasons for pregnant homeless women to receive very limited antenatal care services at health care facilities [15]. Most maternity deaths occur from the first 24 h up to the first week of childbirth; therefore, the world health organization recommended every mother to receive postnatal care service within 24 h even when a mother gave birth at home [35]. However, the finding of this study is inconsistent with the world health organization recommendation in that almost all homeless women do not utilize post-natal care services during their recent post- delivery.

The other factor hindering maternity service utilization among homeless women were religious and traditional beliefs. They believe that maternity health care service is needed only for abnormal pregnancy, sick mother; they strongly believe that there is no need to receive antenatal care, skilled birth attendant and postnatal care because “St. marry” will bless their pregnancy. As supported by an earlier qualitative study conducted in Indonesia some traditional and religious misconceptions about maternity health care services were hindered homeless women from receiving antenatal care services [14]. Participants recall bias might have occurred during interviews. Despite these limitations, this study makes a valuable contribution to the literature a front line study to consider a marginalized and neglected group of the population about whom the experience on maternity health service utilization and challenges they face during pregnancy, childbirth and their post-delivery.

## Conclusions

Though maternity health care service utilization is the most crucial intervention to reduce maternity and newborn deaths, this study shows that most homeless women do not use the most important maternity care services during their pregnancy, delivery and after delivery. Lack of awareness and permanent place, fear of stigma and discrimination, previous maternity care service experience, negligence and lack of social support, religious and traditional beliefs poor socio-economic status and poor approach of health care professionals were found to be the reasons hindering homeless mothers not to access and use maternity care services. Therefore, it needs a great effort and attentions of all the concerned bodies to design and implement appropriate maternity health service information, education and communication strategies to improve maternity health care service utilization.

## Data Availability

All data is contained in the manuscript.

## Abbreviations

AIDS: Acquired immune deficiency syndrome
ANC: Antenatal care
DHS: Demographic and Health Survey
FMoH: Federal Ministry of Health
HCW: Health care workers
HEW: Health extension worker
HIV: Human immunodeficiency virus
HW: Homeless Women
NGO: Non-Governmental Organization
PMTCT: Prevention mother to child transmission
PNC: Postnatal care
SBA: Skilled birth attendant
STDs: Sexually transmitted disease
TBA: Traditional birth attendants
WHO: World Health Organization
